# Effects of Groove and Steel Fiber on Shear Properties of Concrete with Recycled Coarse Aggregate

**DOI:** 10.3390/ma13204537

**Published:** 2020-10-13

**Authors:** Danying Gao, Yongming Yan, Yuyang Pang, Jiyu Tang, Lin Yang, Zhiqiang Gu

**Affiliations:** 1 School of Civil Engineering, Zhengzhou University, Zhengzhou 450001, China; gdy@zzu.edu.cn (D.G.); tjy74@zzu.edu.cn (J.T.); 2 School of Water Conservancy Engineering, Zhengzhou University, Zhengzhou 450001, China; yanglin06142@zzu.edu.cn (L.Y.); zqgzzu@163.com (Z.G.); 3 School of Civil Engineering, Henan University of Engineering, No.1, Xianghe Road, Zhengzhou 451191, China

**Keywords:** recycled coarse aggregate, steel fiber, double-side direct shear, shear section height, shear strength

## Abstract

In this paper, a series of shear specimens with or without groove were manufactured to mainly analyze the effects of grooves (or shear section height) and steel fibers on the shear properties of concrete with recycled coarse aggregate through double-side direct shear test. In addition, the relationship between the shear strength and the compressive strength and splitting tensile strength of steel fiber reinforced concrete with recycled coarse aggregate (SFRCAC) was also discussed. The experimental results showed that the peak load, deformation corresponding to the peak load and calculated shear strength of the specimens with grooves were lower than those of the specimens without grooves. The steel fiber and recycled coarse aggregate (RCA) had a significant effect on the shear properties of SFRCAC. As the volume content of steel fibers increased, the shear strength of SFRCAC and the corresponding deformation increased gradually. With the replacement ratio of RCA increasing, the shear strength of SFRCAC decreased but the corresponding deformation increased gradually. Finally, the formula for calculating the shear strength of SFRCAC was proposed by analyzing and fitting the test results and the data of related literature.

## 1. Introduction

The usage of waste concrete as the recycled coarse aggregate (RCA) has become more common in the recent years, which forms the win-win situation by promoting the recycling of construction waste and reducing the environmental pollution caused by the mining of building raw materials [1,2,3,4,5,6]. The RCA obtained by the relevant treatment of the waste concrete is mainly composed of the natural coarse aggregate (NCA) covered with a layer of porous aged cement paste [7], and exhibits the greater roughness, higher water absorption and crushing index compared with the NCA [8,9,10]. These factors lead to some defects in strength, toughness, crack resistance, durability of RCA and bonded interface between cement paste and RCA [11,12]. Therefore, the wide application of RCA in engineering is greatly limited [13]. Existing studies have proved that the steel fiber can not only improve the tensile, flexural and shear properties of the concrete, greatly enhance the fracture toughness, impact resistance and durability of concrete [14,15,16,17], but also been proven very advantageous in fire resistance [18,19,20]. Referring to the previous studies, it may be considered to remedy the shortcomings and to improve the related mechanical properties of recycled concrete by adding a certain amount of steel fibers, which is extremely beneficial to the application of concrete with recycled coarse aggregate in civil engineering [21].

At present, the studies on the mechanical properties of steel fiber reinforced concrete with recycled coarse aggregate (SFRCAC) mainly focus on the basic mechanical properties, such as Ramesh [22] investigated the split-tensile strength, compressive strength, the toughness under compression and the elastic modulus for 25 SFRCAC mixes. In addition, Gao et al. [23,24,25,26] studied the compressive strength, flexural strength, durability and mixture proportion design method by taking the replacement ratio of reclaimed aggregate and the volume content of steel fiber as the influencing parameters. However, there are few studies on the shear properties of SFRCAC. Wang et al. [27,28] studied the shear strength of SFRCAC by using JSCE-SF6 [29] standard test method. The results showed that the shear strength of SFRCAC gradually decreased with increasing content of RCA. Gao et al. [30] investigated the shear properties of SFRCAC in accordance with the method specified in the Chinese Standard (CECS 13:2009) [31]. The test results indicated that the shear strength of SFRCAC increased as the volume content of steel fibers and the compressive strength of matrix increased, while the shear strength of SRFCAC decreased with increasing replacement ratio of RCA. Carnot [32] used the direct shear test method to study the shear properties of plain concrete. Results showed that the height of the shear section of the specimen had a certain impact on the shear properties of concrete. According to the results of the shear properties of fiber reinforced concrete from the direct shear test, Kazemi [33] found that the specimens with grooves of different sizes had a significant influence on the shear properties of fiber reinforced concrete. It can be seen that the direct shear methods are often employed to investigate the shear properties in the above related literatures, and the shear strength is related to the size of groove or the height of the direct shear section. However, to the best of the authors’ knowledge, the systematical studies on the influences of the groove or shear section height, volume content of steel fibers, water cement ratio and replacement ratio of RCA on the shear properties of SFRCAC have not been reported. Therefore, it is necessary to investigate the shear properties of SFRCAC in the aspects of its component materials and the change of shear section height by using the specimens with or without groove. In addition, the usage of SFRCAC is increasing in recent years, it is of great significance to study the shear properties of SFRCAC for its extensive application in engineering.

In this paper, the double-sided direct shear method recommended in the Chinese Standard (CECS 13:2009) was used to investigate the effects of the volume content of steel fibers, water cement ratio and replacement ratio of RCA on the shear properties of SFRCAC. Especially, the shear specimen with grooves was applied to further analyze the influence of shear section height on the shear properties of SFRCAC. Finally, the formula for calculating the shear strength of SFRCAC was obtained by fitting the experimental data and those from the related literature.

## 2. Experimental Program

### 2.1. Materials

**Figure 1:**
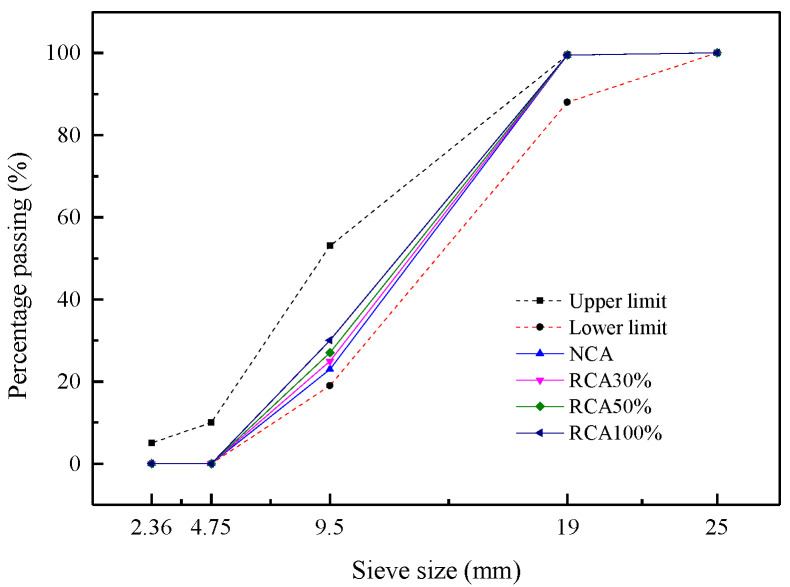
Particle size distribution of coarse aggregates.

**Figure 2:**
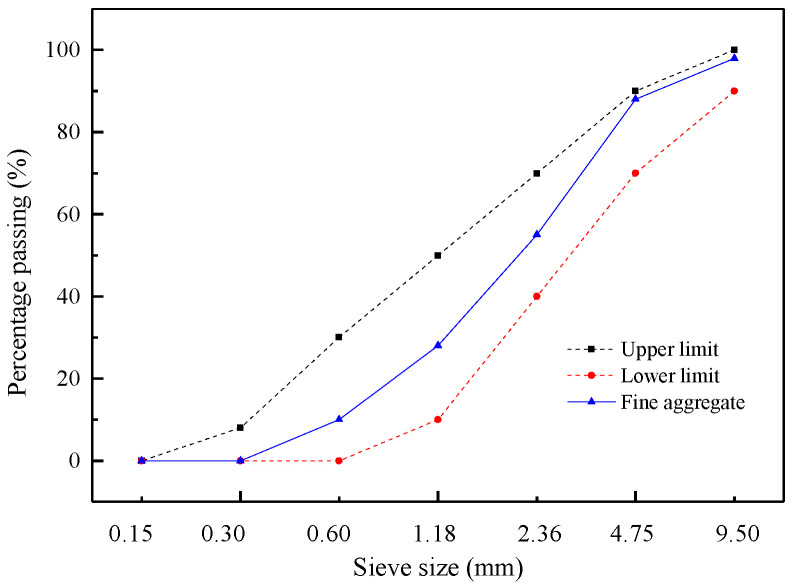
Particle size distribution of fine aggregate.

**Figure 3:**
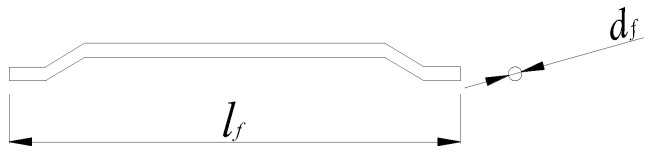
Steel fiber.

The cement used in this study was P.O 42.5 ordinary Portland cement. The coarse aggregate consisted of the recycled coarse aggregate (RCA) obtained from the waste concrete and natural coarse aggregate (NCA). The waste concrete had a compressive strength of 30–50 MPa, and should be crushed before using. Among them, the particles with a size range of 4.75 mm to 20 mm were selected as the RCA. In addition, the limestone gravels with a particle size of 4.75–20 mm were selected as the NCA in this study. The physical and mechanical properties of RCA and NCA were tested according to the Chinese Standard GB/T 25177–2010 [34]. The measured physical properties of coarse aggregate are shown in Table 1. The particle size distribution of coarse aggregates is depicted in Figure 1. It can be seen from the Table 1 that compared with NCA, the RCA exhibited the lower apparent density, larger porosity and water absorption due to the old cement paste adhered on the surface, which had many micro cracks and holes. The fine aggregate was river sand, which had a fineness modulus of 2.68 and an apparent density of 2552 kg/m^3^. The particle size distribution of fine aggregate is shown in Figure 2. The hook shaped steel fibers as shown in Figure 3, produced by Shanghai Bekaert No.2 Steel Limited Company (Shanghai, China). were adopted, which had a average length (*l_f_*) of 35 mm, a nominal diameter (*d_f_*) of 0.55 mm, a tensile strength of 1345 N/mm^2^, and an aspect ratio (*l_f_/d_f_*) of 63. The properties of steel fibers are listed in Table 2. A high-performance water reducing agent was used to reduce the unit water demand of concrete, and to improve the workability of concrete, the dosage of which was about 1% of cement by weight. The measured water reduction rate of superplasticizer was 25%.

### 2.2. Test Parameters and Mixture Proportions

In order to systematically investigate the effects of volume content of steel fibers (*V_f_*), height of shear section, water cement ratio (*W/C*) and replacement ratio of RCA (*r_g_*) on the shear properties of SFRCAC, the test plan was developed according to the principle that one effecting factor changed while other factors remained unchanged, as shown in Table 3. The specimen number was composed of letters and numbers. The first letter represented whether the specimen was grooved; the second letter and the first number represented the concrete grade, which corresponded to different water-cement ratios; the third letter and the second number represented the replacement ratio of RCA; the last letter and the last number represented the volume content of steel fibers. For example, N-C45R50F1 represented that the shear specimen was not grooved; the water cement ratio was 0.4, the replacement ratio of RCA was 50% and the volume content of steel fibers was 1.0%. A total of eighty four shear specimens were manufactured for testing, which were divided into twenty one groups. The mixture proportions of each type concrete are listed in Table 4.

**Figure 4:**
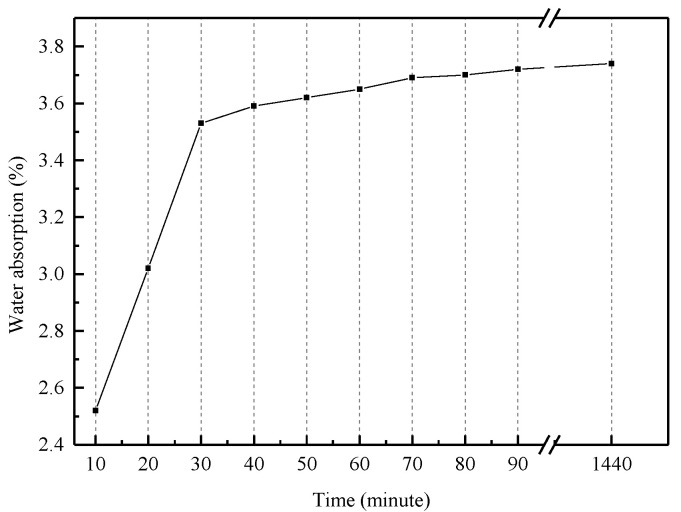
Water absorption curve of recycled coarse aggregate over time.

In this study, the amount of each material could be calculated in accordance with the Chinese Standard (CECS 13:2009). Considering the influence of larger water absorption of RCA, it was necessary to pre-wet the RCA before mixing according to the Chinese Standard GB/T 25177-2010. In order to eliminate the uncertainty of measurement, the recycled coarse aggregate should be placed in the drying box for 24 h before the water absorption test. The pre-wetting water quantity was determined according to the measured water absorption of RCA. The measured water absorption curve of recycled coarse aggregate over time is shown in Figure 4. It could be seen that the amount of water absorption of RCA increased linearly in the first 30 min, and the maximum water absorption was reached after 24 h of pre-wetting. In order to shorten the mixing time, the pre-wetting water consumption was determined by the water absorption at 30 min, the main reason was that the water absorption of RCA had reached 94.4% of 24 h at 30 min.

### 2.3. Specimen Preparation

The concrete used in this study was manufactured by using the horizontal mixer, and all specimens for the test were poured according to the Chinese Standard (CECS 13:2009) and GB/T 50080-2016 [31,35], which had a standard size of 100 mm × 100 mm × 300 mm. Four specimens were prepared for each type of SFRCAC and the average value of the measured shear strength of the four specimens was taken as the measurement result. If the difference between the maximum or minimum value of the four measured values and the mean value of the two intermediate values was greater than 15% of the mean value, then the mean value of the two intermediate values was taken as the shear strength of this group of specimens. If the difference between the two samples and the mean value was greater than 15%, the test results of this group of specimens were invalid. Meanwhile, six standard cube samples with a size of 150 mm × 150 mm × 150 mm were prepared to test the compressive strength and splitting strength of the corresponding type of SFRCAC. All specimens should be vibrated on the shaking table. Subsequently, each specimen was removed to the curing room with a relative humidity of about 95% and a temperature of 20 °C for 28 days.

### 2.4. Test Procedures

**Figure 5:**
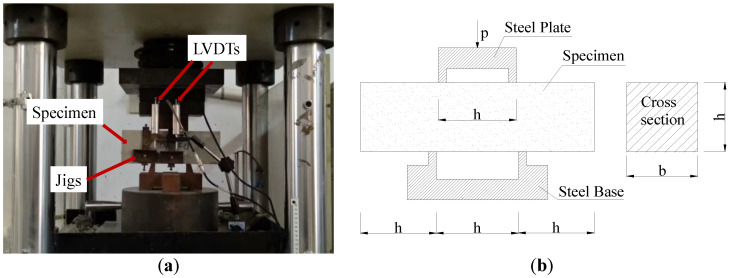
Shear strength test: (**a**) Photo of the shear strength test and (**b**) Schematic representation.

**Figure 6:**
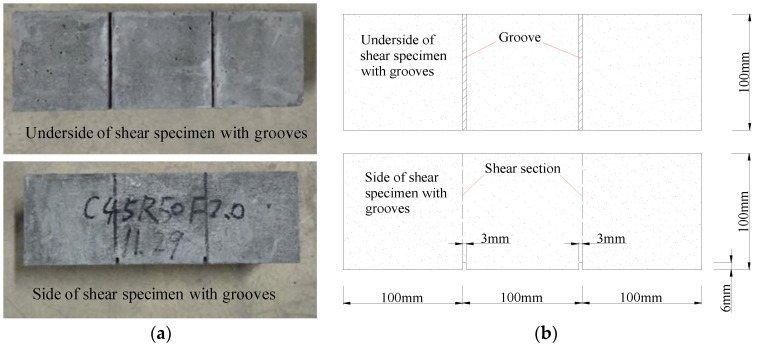
Shear test specimen with grooves: (**a**) Photo of specimen and (**b**) Schematic representation.

All specimens were tested on 3000 kN electro-hydraulic servo pressure tester. Refered to the Chinese Standard (CECS 13:2009), the double-sided direct shear specimens were manufactured to investigate the shear properties of SFRCAC, and the specimens were loaded with the displacement control at a rate of 0.3 mm/min until the deformation reached 3 mm, as shown in Figure 5. For the experimental groups investigating the impact of height of shear section on the shear properties of SFRCAC, each specimen was grooved in the lower part of two shear sections, and the grooves had a width of 3 mm and a depth of 6 mm. The diagram of shear test specimen with grooves is shown in Figure 6.

## 3. Test Results and Analysis

### 3.1. Shear Failure Mode

#### 3.1.1. Shear Failure Mode of the Specimens without Steel Fibers

**Figure 7:**
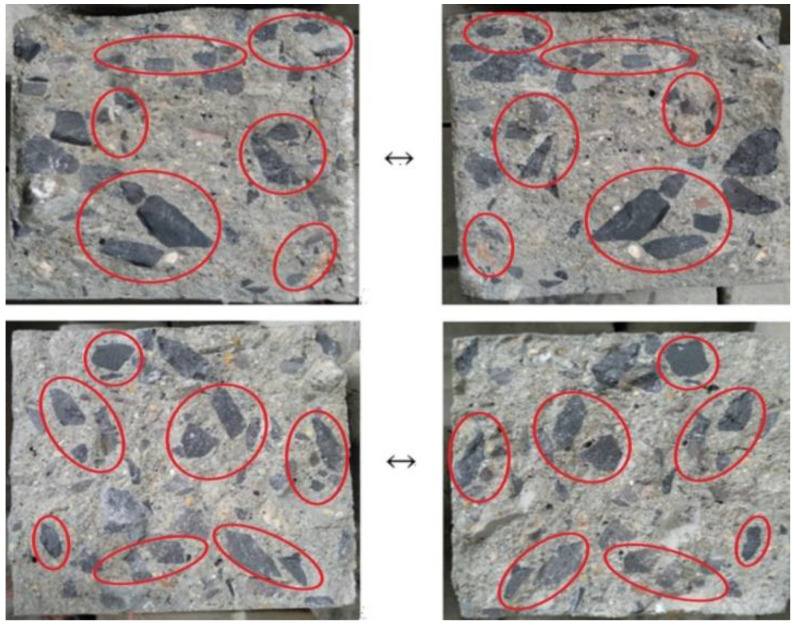
Failure mode of shear specimen without steel fibers.

For the specimens without steel fibers, the significant brittle failure could be observed for each specimen. Some visible tiny cracks occurred along the shear sections immediately and then fracture happened rapidly as the load increased to a certain value. Subsequently, the specimens were cut into three parts along the two shear sections when the failure occurred. Figure 7 presents the failure diagram of two shear sections of shear specimen without steel fibers. It can be easily found that the shear failure section was relatively smooth and the fracture surface passed through the RCA. The main reason was that the strength of RCA was lower than that of cement matrix, which led to the shear cracks passing through the RCA directly. It was mentioned in relevant literature that there were two weak links in RCA, which were the inherent cracks produced in crushing and the stone-mortar interface on RCA, and the failure of RCA basically originated from these weak points [7].

#### 3.1.2. Shear Failure Mode of the Specimens with Steel Fibers

**Figure 8:**
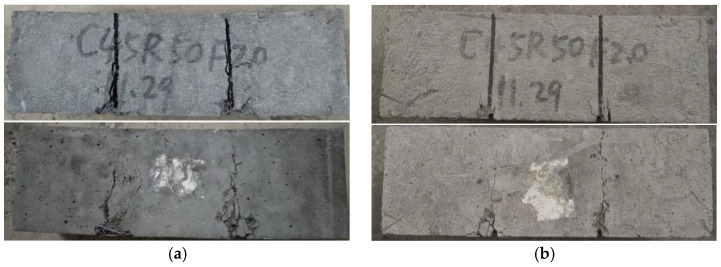
Failure mode of shear specimen with steel fibers. (**a**) Shear specimen without groove. (**b**) Shear specimen with groove.

For the specimens with steel fibers, the typical ductile failure could be observed for each specimen. The first visible fine crack appeared as the load increased to a certain value. With increasing load, abundant micro-cracks appeared near the first crack. Subsequently, the cracks further extended, and finally formed a shear fracture zone through the shear failure section. With the increase of shear deformation, some steel fibers were gradually pulled out. In this process, the sound of steel fibers pulled out from cement matrix could be heard. Meanwhile, it could be found that the cement matrix peeled off from the specimen surface, and the lower volume contents of steel fibers, the more serious the spalling of surface concrete. The failure diagram of shear specimens with steel fibers is shown in Figure 8. It can be observed that the shear specimens with or without grooves were not cut into three parts due to the bridging effect of steel fibers.

### 3.2. Shear Load-Deformation Curves

#### 3.2.1. Shear Load-Deformation Curves of Specimens without Grooves

**Figure 9:**
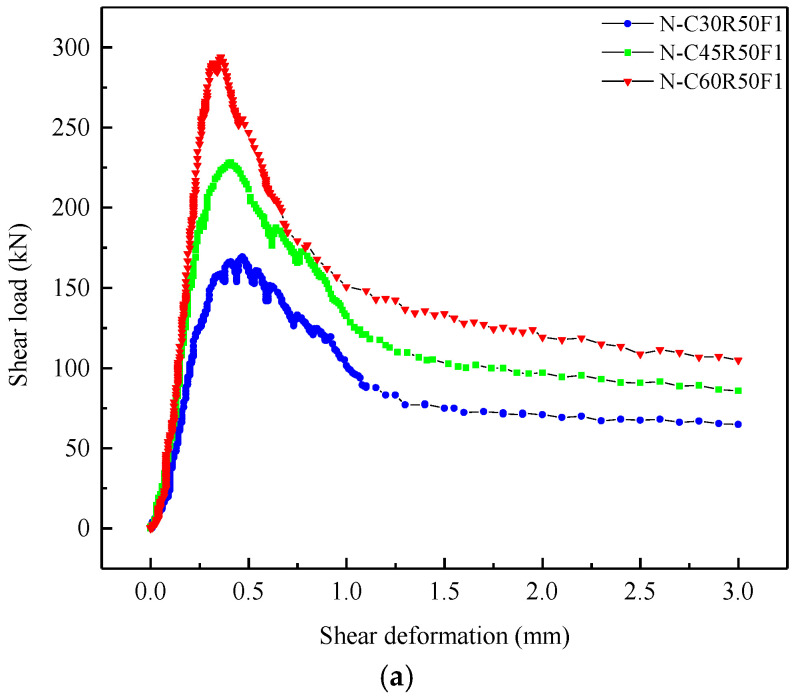

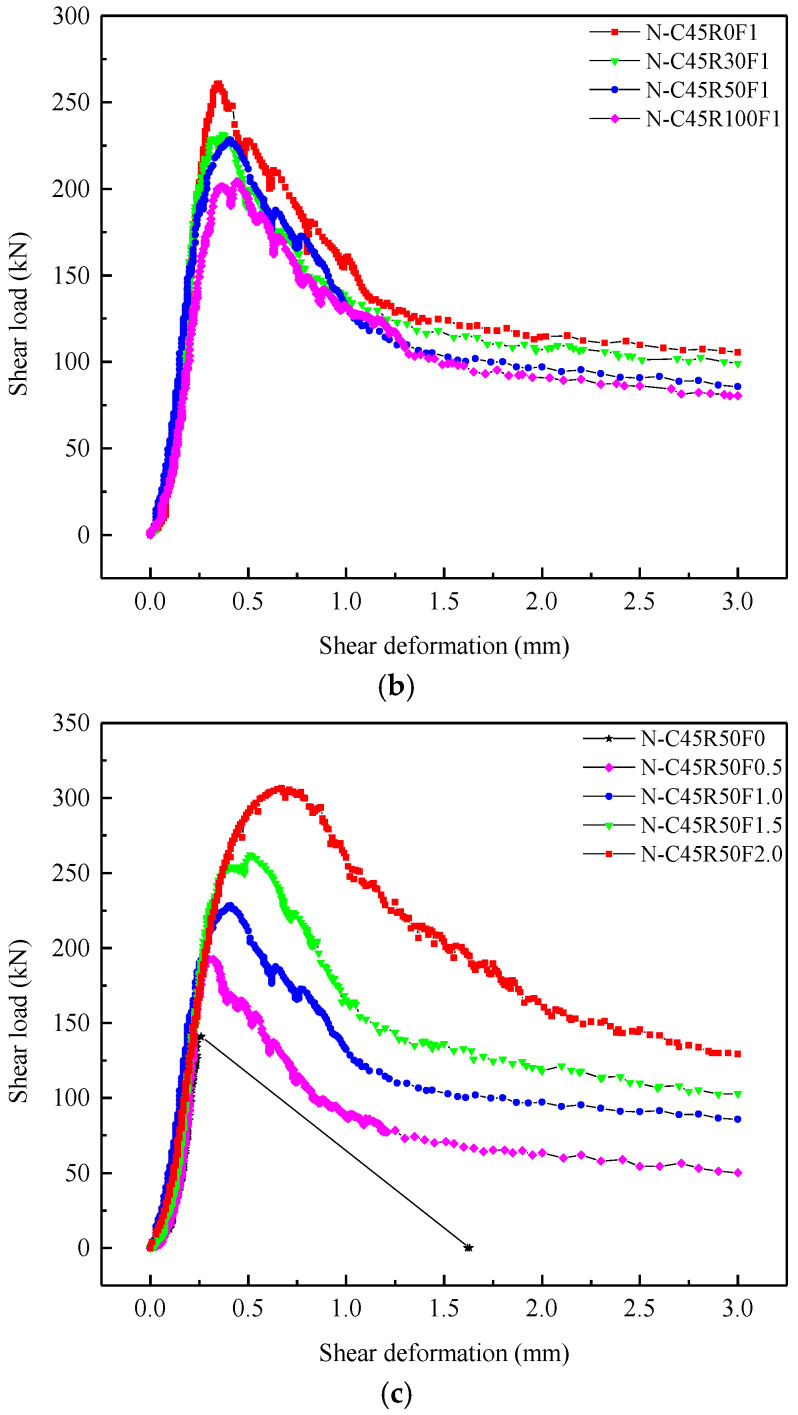
Shear load-deformation curves of specimens without grooves under different (**a**) water cement ratio; (**b**) replacement ratio of RCA; (**c**) volume content of steel fibers.

Figure 9 plots the shear load-deformation curves of specimens without grooves under different parameters. Generally, the shear load-deformation curve of each specimen could be divided into three stages: (1) in the elastic stage, the shear load increased linearly with increasing of deformation; (2) in the elastic-plastic stage, the shear load increased nonlinearly as the deformation increased until the maximum shear load was reached; (3) in the softening stage, the shear load decreased gradually with increasing deformation until the deformation reached a certain value, then the shear load was almost constant.

Figure 9a reveals the effects of *W/C* on the shear load-deformation curves. For this series of specimens, *W/C* varied from 0.3 to 0.55, while *r_g_* and *V_f_* were fixed at 50% and 1.0%, respectively. It can be observed that the peak shear load of SFRCAC increased gradually with the decrease of *W/C*, while the deformation corresponding to peak load decreased with the decrease of *W/C*. In the elastic stage, the slope of curve increased as *W/C* decreased, which indicated that the shear brittleness of SFRCAC increased with the decrease of *W/C*. In the softening stage, the absolute value of the curve slopes decreased as *W/C* increased, which indicated that SFRCAC showed the significant strain softening after the peak load was reached. The main reason was that SFRCAC had the higher strength, the greater brittleness and the smaller toughness for the specimen with the smaller *W/C*.

Figure 9b presents the effects of the replacement ratio of RCA on the shear load-deformation curves. For this series of specimens, *r_g_* varied from 0% to 100%, and *W/C* and *V_f_* were fixed at 0.4 and 1.0%, respectively. The peak shear load of SFRCAC decreased gradually with increasing *r_g_*, while the deformation corresponding to peak load increased as *r_g_* increased. In the elastic stage, the slope of curve decreased as *r_g_* increased, which indicated that the shear brittleness of the SFRCAC decreases with increasing *r_g_*. In the softening stage, the absolute value of the curve slopes decreased as increasing *r_g_*, which indicated that SFRCAC showed the obvious strain softening after the peak load was reached. The main reason was that the RCA had many micro cracks and lower strength.

Figure 9c shows the effects of *V_f_* on the shear load-deformation curves. For this series of specimens, *V_f_* varied from 0% to 2.0%, and *W/C* and *r_g_* were fixed at 0.4 and 50%, respectively. The peak shear load of SFRCAC and the deformation corresponding to peak load increased gradually with increasing *V_f_*. In the elastic stage, the slope of curve decreased as *V_f_* increased, which indicated that the shear brittleness of the SFRCAC decreased with increasing *V_f_*. In the softening stage, as *V_f_* increased, the absolute value of the curve slopes decreased as increasing *V_f_*, which indicated that SFRCAC showed the obvious strain softening after the peak load was reached. The main reason was that the steel fiber passing through the crack played an important role in resisting tensile and shear stress after the cracks appeared.

#### 3.2.2. Shear Load-Deformation Curves of Specimens with Grooves

**Figure 10:**
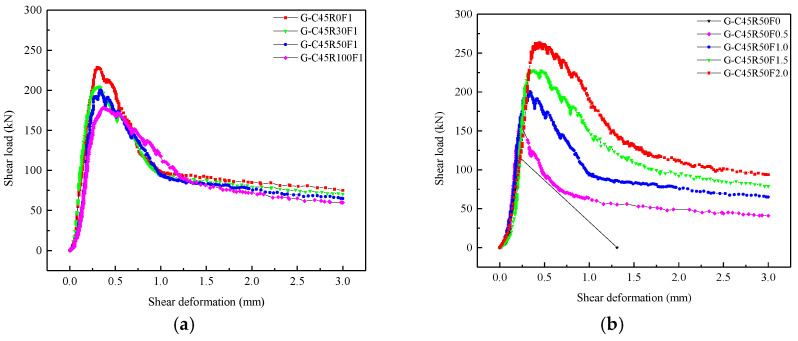
Shear load-deformation curves of specimens with grooves under different (**a**) replacement ratio of RCA; (**b**) volume content of steel fibers.

Figure 10 shows the shear load-deformation curves of specimens with grooves under different *r_g_* and *V_f_*. Generally, the shear load-deformation curve of each specimen with grooves can also be divided into three stages: (1) the elastic stage; (2) the elastic-plastic stage; and (3) the softening stage. It could be observed that the development rule of curves for specimens with grooves was consistent with that of the specimen without grooves. The difference was that the peak loads of specimens with grooves were smaller, and the deformations corresponding to peak loads were also smaller.

### 3.3. Shear Strength

According to the Chinese Standard (CECS 13:2009), the shear strength of the specimen in this study can be calculated by Equations (1)–(3).

(1) $$f_{fv}=F_{\max}/2bh$$

(2) $$b=(b_{1}+b_{2}+b_{3}+b_{4})/4$$

(3) $$h=(h_{1}+h_{2}+h_{3}+h_{4})/4$$

where, *f_fv_* is the ultimate shear strength of SFRCAC; *F_max_* is the peak shear load; *b* is calculated by using Equation (2); *b*_1_, *b*_2_, *b*_3_ and *b*_4_ are four widths measured on two predetermined failure sections, respectively; *h* is calculated by using Equation (3); *h*_1_, *h*_2_, *h*_3_ and *h*_4_ are four heights measured on two predetermined failure sections, respectively. The test results and calculated shear strength of SFRCAC are shown in Table 5.

#### 3.3.1. Influence of *W/C*, *r_g_* and *V_f_* on the Shear Specimens without Grooves

**Figure 11:**
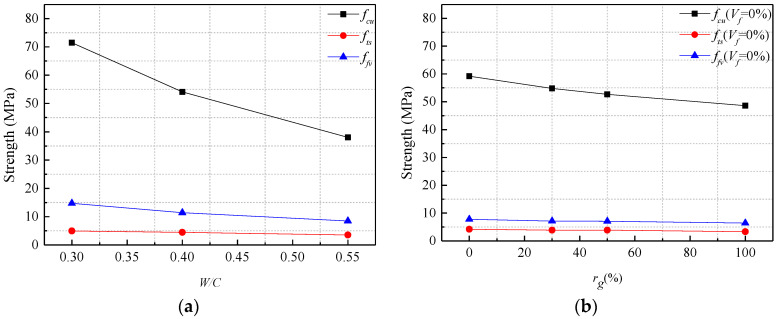

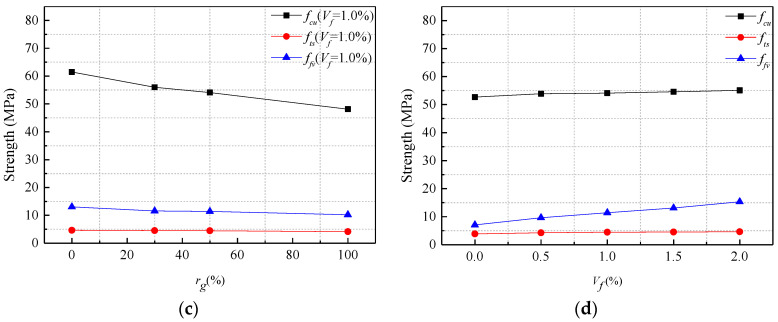
Effects of (**a**) *W/C*; (**b**) *r_g_*; (**c**) *r_g_*; (**d**) *V_f_* on the strengths of SFRCAC specimens without grooves.

The average cube compressive strength (*f_cu_*), splitting tensile strength (*f_ts_*), shear strength (*f_fv_*), shear-compression ratio (*f_fv_/f_cu_*), and shear-tensile ratio (*f_fv_/f_ts_*) of SFRCAC specimens are shown in Table 5. Figure 11a shows the varieties of *f_cu_*, *f_ts_* and *f_fv_* with increasing *W/C*. With the range of *W/C* varying from 0.3 to 0.55, *f_cu_*, *f_ts_*, *f_fv_* and *f_fv_/f_ts_* of SFRCAC decreased gradually, *f_fv_/f_cu_* basically remained unchanged. The reason for above results was that with the increasing *W/C*, the matrix strength of SFRCAC decreased.

Figure 11b,c show the varieties of *f_cu_*, *f_ts_* and *f_fv_* with increasing *r_g_*. For this series of specimens, the volume contents of steel fibers were 0% and 1.0%, respectively. With the range of *r_g_* varying from 0% to 100%, *f_cu_*, *f_ts_* and *f_fv_* of SFRCAC decreased gradually, while *f_fv_/f_ts_* was almost unchanged. As the reduction percentages of *f_fv_* and *f_cu_* were close, it could be observed that when *V_f_* was constant and *r_g_* changed, *f_fv_/f_cu_* was nearly a fixed values. The reason was that many micro cracks in RCA resulted in the low strength of RCA, which reduced the strength of SFRCAC.

Figure 11d shows the varieties of *f_cu_*, *f_ts_* and *f_fv_* with increasing *V_f_*. With the range of *V_f_* varying from 0% to 2.0, *f_ts_*, *f_fv_*, *f_fv_/f_cu_* and *f_fv_/f_ts_* of SFRCAC increased gradually. The splitting tensile strength increased from 3.85 MPa to 4.63 MPa, which increased by 20.30%. The shear strength increased from 7.05 MPa to 15.32 MPa, which increased by 117.30%. The ratios of shear strength to splitting tensile strength were 1.831, 2.271, 2.553, 2.894 and 3.309 respectively. The reason was that the steel fiber played an important role to resist the tensile and shear stress after the cracks appeared, which made the cracked concrete still had a certain bearing capacity. Hence, with the increase of *V_f_*, the shear strength of the SFRCAC increased. However, *f_cu_* was almost unchanged as *V_f_* increased. The same conclusion could be found in the relevant literature, which indicated that the steel fiber had little effect on *f_cu_* of concrete [14,36].

#### 3.3.2. Influence of *r_g_* and *V_f_* on Shear Strength of Specimens with Grooves

**Figure 12:**
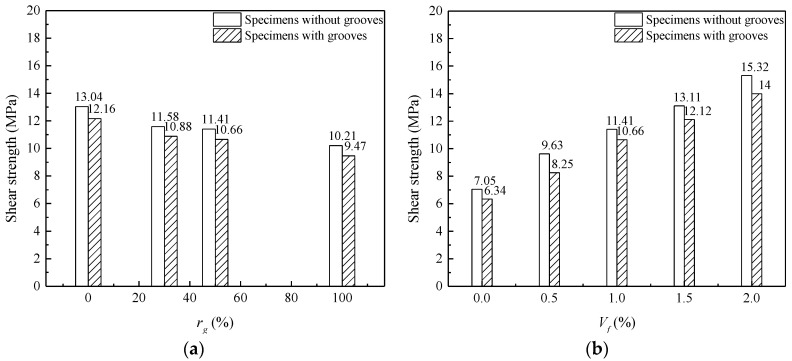
Effects of (**a**) *r_g_*; (**b**) *V_f_* on the strengths of SFRCAC specimens with grooves.

Figure 12 shows the effects of *r_g_* and *V_f_* on the strengths of SFRCAC specimens with grooves. Compared the Figure 12 with Figure 11, the same development trend of shear strength could be observed for specimens with or without grooves as *r_g_* and *V_f_* increased.

Figure 12a presents the effects of *r_g_* on the shear strength of SFRCAC specimens with grooves. As *r_g_* increased from 0% to 100%, the shear strengths of specimens with grooves were 8.74%, 6.04%, 6.57% and 7.25% lower than that of specimens without grooves, respectively. Figure 12b shows the effects of *V_f_* on the shear strength of SFRCAC specimens with grooves. As *V_f_* increased from 0% to 2%, the shear strengths of specimens with grooves were 10.07%, 14.33%, 6.57%, 7.55% and 8.62% lower than that of specimens without grooves, respectively. The above test results can be analyzed from two aspects: (1) the steel fiber and coarse aggregate in the grooved part of the shear specimen were cut off; (2) the magnitude and distribution of the stresses on the shear section of specimens with grooves had changed. For the specimen with grooves, the value of *h* changed from 100 mm to 94 mm and the area of shear section decreased by 6%. Therefore, the shear strengths of the specimen with grooves were 6% less than that of the specimen without grooves. From the above analysis, we can obtain Equations (4)–(7).

(4) τ=F/2bh

(5) $$\tau_{1}=F_{1}/2bh_{1}$$

(6) $$\tau_{1}<\tau \left(1-6\%\right)$$

(7) $$F_{1}<88.36F$$

where, *F*_1_, *h*_1_, and *τ*_1_ are the ultimate load, height and shear strength of specimen with grooves, respectively. *F*, *h*, and *τ* are the ultimate load, height and shear strength of specimen without grooves, respectively.

From the above analysis, it could be concluded that the reduction ratio of the ultimate shear load of specimen with grooves was more than 11.64% compared with the specimen without grooves. The reason may be related to the stress mechanism of the shear specimen in the double-sided direct shear test. The experimental study and theoretical analysis showed that the shear strength obtained by double-sided direct shear test was an approximate value of pure shear strength. The double-sided direct shear test could be regarded as a special case that the shear span ratio approached to zero when the simply supported beam was loaded at four points. In this case, the types of stress in the shear section could be divided into horizontal normal stress, vertical shear stress and vertical normal stress. Therefore, the combined force of vertical shear stress and vertical normal stress in the shear section was the ultimate shear load *F* obtained by the test machine. The vertical shear stress changed parabola from top to bottom along the shear section and reached the maximum value in the middle of the shear section height, and the vertical normal stress produced by extrusion was not evenly distributed on the shear section, but gradually decreased from top to bottom along the shear section height [37]. According to the above theoretical basis, the reason why the maximum shear load and average shear strength of the specimen with grooves decrease by more than a certain percentage compared with the specimen without grooves might be qualitatively explained.

According to the above research results, the effect of groove on the shear strength of SFRCAC was obvious under the direct shear condition. Since this experiment did not specifically study the influence of different groove depths on the shear strength of SFRCAC, it was necessary to consider the influence of this change factor on the shear strength of SFRCAC in the future research steps.

#### 3.3.3. Calculation Method of Shear Strength of SFRCAC

Existing researches showed that the influences of steel fiber on the strength and toughness of concrete were mainly related to the length-diameter ratio (*l_f_*/*d_f_*) and volume content of steel fiber (*V_f_*) [20,38,39,40]. In this study, parameter *λ_f_* was introduced to investigate the effect of steel fiber on the properties of SFRCAC, which could be calculated by Equation (8).

(8) $$\lambda_{f}=V_{f}\left(l_{f}/d_{f}\right)$$

**Figure 13:**
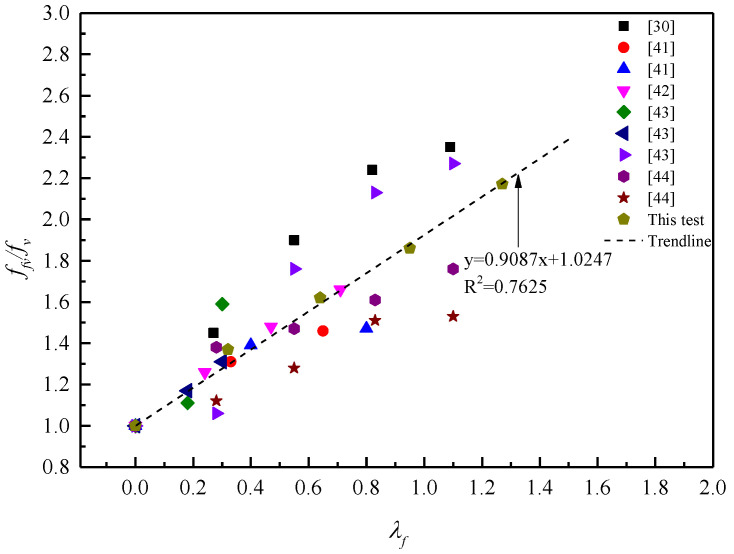
Relationship between *f_fv_/f_v_* and *λ_f_*.

Figure 13 presents the relationship between *f_fv_/f_v_* and *λ_f_* from the test results in related references and this study [30,41,42,43,44], where *f_fv_/f_v_* is the ratio of shear strength between steel fiber reinforced concrete and plain concrete with the same mixture proportions, it can be seen that *f_fv_*/*f_v_* increases almost linearly with increasing *λ_f_*. Therefore, the shear strength of SFRCAC can be calculated by Equation (9):

(9) $$f_{fv}=f_{v}(1+0.9\lambda_{f})$$

where, *f_fv_* is the shear strength of SFRCAC; *f_v_* is the shear strength of plain concrete with the same mix ratio as that of SFRCAC; *λ_f_* is the characteristic parameter of steel fiber content.

In addition, this study and existing researches [45,46,47] showed that *r_g_* had little effect on *f_fv_/f_cu_*. Therefore, the calculation model between cube compressive strength and shear strength of plain concrete could be applicable to concrete with recycled coarse aggregate, and the relationship between cube compressive strength and shear strength of plain concrete could be expressed by Equation (10).

(10) $$f_{v}=0.8f_{cu}^{0.55}$$

However, the results of this study and existing researches [14,30,36] indicated that *f_fv_/f_cu_* was nearly a fixed value for the SFRCAC specimen with the same *V_f_*, steel fiber had little effect on *f_cu_* of concrete. The expression of the shear strength of SFRCAC could be obtained as Equation (11) by substituting Equation (10) into Equation (9).

(11) $$f_{fv}=0.8f_{cu}^{0.55}(1+0.9\lambda_{f})$$

Table 6 lists the test values and predicted values of the shear strength for each specimen. The average ratio of the experimental values to the predicted values and the coefficient of variation were 0.999 and 5.09%, respectively, which indicated that the shear strength of SFRCAC can be predicted by Equation (11).

## 4. Conclusions

In this paper, the effects of grooves (or shear section height) and steel fibers on the shear properties of concrete with recycled coarse aggregate were investigated by double-side direct shear test. According to the test results and analysis, the following conclusions can be obtained.

1. The shear strength and shear deformation of specimens with grooves were lower than those of specimens without grooves under the same mixture of SFRCAC. Among them, the height of the shear section decreased by 6%, and the shear strength decreased by more than 6%. For the shear specimens with grooves, the influence of the replacement ratio of RCA and volume content of steel fibers on shear strength and shear deformation had the same trend as that of shear specimens without grooves. According to the above research results, it could be concluded that the effect of groove on the shear strength of SFRCAC was obvious. It was necessary to consider the influence of this change factor on the shear strength of SFRCAC in the future research steps.
2. For specimens with the fixed water cement ratio and replacement ratio of RCA, with the increase of the volume content of steel fibers, the shear strength, shear deformation corresponding to peak load and shear toughness of SFRCAC increased gradually. The shear strength of SFRCAC increased by 117.30% as the volume content of steel fibers increased from 0% to 2.0%.
3. With the increase of water cement ratio, the shear strength of SFRCAC decreased, while the shear deformation corresponding to peak load and shear toughness increased gradually. The shear strength of SFRCAC decreased by 42.38% as the water cement ratio increased from 0.3 to 0.55.
4. For specimens with the fixed water cement ratio and volume content of steel fibers, with the increase of the replacement ratio of RCA, the shear strength of SFRCAC decreased, while the shear deformation corresponding to peak load and shear toughness increased gradually. The shear strength of SFRCAC decreased by 21.70% as the replacement ratio of RCA increased from 0% to 100%.

## Figures and Tables

**Table 1:** Physical properties of coarse aggregate.

Aggregate Type	Apparent Density (kg/m^3^)	Loose Bulk Density (kg/m^3^)	Water Absorption (%)	Crush Index (%)	Porosity (%)
NCA	2805	1552	1.35	8.9	42.3
RCA	2650	1375	4.49	14.5	49.2

**Table 2:** Properties of steel fibers.

*d_f_* (mm)	*l_f_* (mm)	*l_f_*/*d_f_*	Tensile Strength (MPa)
0.55	35	63.64	1345

**Table 3:** Test plan.

Group	*W/C*	*r_g_*	*V_f_*
N-C30R50F1	0.55	50%	1.0%
N-C45R50F1	0.4	50%	1.0%
N-C60R50F1	0.3	50%	1.0%
N-C45R0F0	0.4	0%	0%
N-C45R30F0	0.4	30%	0%
N-C45R50F0	0.4	50%	0%
N-C45R100F0	0.4	100%	0%
N-C45R0F1	0.4	0%	1.0%
N-C45R30F1	0.4	30%	1.0%
N-C45R50F1	0.4	50%	1.0%
N-C45R100F1	0.4	100%	1.0%
N-C45R50F0	0.4	50%	0%
N-C45R50F0.5	0.4	50%	0.5%
N-C45R50F1	0.4	50%	1.0%
N-C45R50F1.5	0.4	50%	1.5%
N-C45R50F2.0	0.4	50%	2.0%
G-C45R0F1	0.4	0%	1.0%
G-C45R30F1	0.4	30%	1.0%
G-C45R50F1	0.4	50%	1.0%
G-C45R100F1	0.4	100%	1.0%
G-C45R50F0	0.4	50%	0%
G-C45R50F0.5	0.4	50%	0.5%
G-C45R50F1	0.4	50%	1.0%
G-C45R50F1.5	0.4	50%	1.5%
G-C45R50F2.0	0.4	50%	2.0%

Note: The letter N indicated that the specimen had not been grooved. The letter G indicated that the specimen had been grooved.

**Table 4:** Summary of concrete mixture proportions.

Group	*W/C*	Water (kg/m^3^)	Cement (kg/m^3^)	NCA (kg/m^3^)	RCA (kg/m^3^)	Fine Aggregate (kg/m^3^)	Steel Fiber (kg/m^3^)	Water Reducer (kg/m^3^)	Additional Water (kg/m^3^)
N-C30R50F1	0.55	166	302	540	540	884	78	3.02	19.06
N-C45R50F1	0.40	166	415	512	512	839	78	4.15	18.08
N-C60R50F1	0.30	166	553	479	479	783	78	5.53	16.89
N-C45R0F0	0.40	166	415	1024	0	839	0	4.15	0
N-C45R30F0	0.40	166	415	717	307	839	0	4.15	10.85
N-C45R100F0	0.40	166	415	0	1024	839	0	4.15	36.16
N-C45R0F1	0.40	166	415	1024	0	839	78	4.15	0
N-C45R30F1	0.40	166	415	717	307	839	78	4.15	10.85
N-C45R100F1	0.40	166	415	0	1024	839	78	4.15	36.16
N-C45R50F0	0.40	166	415	512	512	839	0	4.15	18.08
N-C45R50F0.5	0.40	166	415	512	512	839	39	4.15	18.08
N-C45R50F1.5	0.40	166	415	512	512	839	117	4.15	18.08
N-C45R50F2.0	0.40	166	415	512	512	839	156	4.15	18.08
G-C45R0F1	0.40	166	415	1024	0	839	78	4.15	0
G-C45R30F1	0.40	166	415	717	307	839	78	4.15	10.85
G-C45R50F1	0.40	166	415	512	512	839	78	4.15	18.08
G-C45R100F1	0.40	166	415	0	1024	839	78	4.15	36.16
G-C45R50F0	0.40	166	415	512	512	839	0	4.15	18.08
G-C45R50F0.5	0.40	166	415	512	512	839	39	4.15	18.08
G-C45R50F1.5	0.40	166	415	512	512	839	117	4.15	18.08
G-C45R50F2.0	0.40	166	415	512	512	839	156	4.15	18.08

**Table 5:** Main test results and calculated shear strength.

Specimen No.	*f_cu_* (MPa)	*f_ts_* (MPa)	Peak Load (kN)	Peak Deformation (mm)	*f_fv_* (MPa)	*SD*	*f_fv_/f_cu_*	*f_fv_/f_ts_*
N-C30R50F1	38.0	3.53	169.32	0.47	8.47	0.43	0.223	2.399
N-C45R50F1	54.1	4.47	228.22	0.41	11.41	0.48	0.211	2.553
N-C60R50F1	71.5	4.94	293.98	0.36	14.70	0.68	0.206	2.976
N-C45R0F0	59.2	4.20	155.31	0.24	7.77	0.41	0.131	1.850
N-C45R30F0	54.8	3.87	142.66	0.25	7.13	0.38	0.130	1.842
N-C45R100F0	48.6	3.32	137.81	0.28	6.39	0.29	0.131	1.925
N-C45R0F1	61.5	4.59	260.73	0.35	13.04	0.56	0.212	2.841
N-C45R30F1	56.0	4.53	231.57	0.37	11.58	0.57	0.207	2.556
N-C45R100F1	48.1	4.12	204.29	0.44	10.21	0.44	0.212	2.478
N-C45R50F0	52.7	3.85	140.93	0.26	7.05	0.37	0.134	1.831
N-C45R50F0.5	53.9	4.24	192.52	0.32	9.63	0.52	0.179	2.271
N-C45R50F1.5	54.6	4.53	262.12	0.51	13.11	0.66	0.240	2.894
N-C45R50F2.0	55.1	4.63	306.44	0.67	15.32	0.75	0.278	3.309
G-C45R0F1	61.5	4.59	228.52	0.30	12.16	0.53	0.198	2.649
G-C45R30F1	56.0	4.53	204.54	0.32	10.88	0.47	0.194	2.402
G-C45R50F1	54.1	4.47	200.37	0.34	10.66	0.41	0.197	2.385
G-C45R100F1	48.1	4.12	177.98	0.37	9.47	0.37	0.197	2.299
G-C45R50F0	52.7	3.85	119.11	0.2	6.34	0.33	0.120	1.647
G-C45R50F0.5	53.9	4.24	155.12	0.25	8.25	0.41	0.153	1.946
G-C45R50F1.5	54.6	4.53	227.86	0.38	12.12	0.52	0.222	2.675
G-C45R50F2.0	55.1	4.63	263.25	0.44	14.00	0.56	0.254	3.024

Note: The letters SD indicated the standard deviation in ffv of the specimen. According to the statistical analysis of SD, the value of SD in each group was very small, and the discrete type of the experimental results was very small. In addition, by analyzing the statistical results of each parameter on the shear properties of SFRCAC, it could be seen that the SD had little influence on the shear properties of each parameter in the test and hardly changed the variation trend of the above parameters on the test results.

**Table 6:** Comparison of calculated values of shear strength with experimental values.

Group	*f_cu_* (MPa)	*λ_f_*	*f_fv_* (MPa)	*f_fv1_* (MPa)	*f_fv_*/*f_fv1_*
N-C30R50F1	38.0	0.636	8.47	9.30	1.10
N-C45R50F1	54.1	0.636	11.41	11.30	0.99
N-C60R50F1	71.5	0.636	14.70	13.17	0.90
N-C45R0F1	61.5	0.636	13.04	12.12	0.93
N-C45R30F1	56.0	0.636	11.58	11.51	0.99
N-C45R100F1	48.1	0.636	10.21	10.59	1.04
N-C45R50F0	52.7	0	7.05	7.08	1.00
N-C45R50F0.5	53.9	0.318	9.63	9.22	0.96
N-C45R50F1.5	54.6	0.954	13.11	13.42	1.02
N-C45R50F2.0	55.1	1.272	15.32	15.56	1.02
N-C45R0F0	59.2	0	7.77	7.55	0.97
N-C45R30F0	54.8	0	7.13	7.23	1.01
N-C45R100F0	48.6	0	6.39	6.77	1.06
Average value					0.999
Coefficient of variation					5.09%

Note: *f_fv_* is the test data, which is calculated by Equation (1). *f_fv1_* is the calculated value, which is calculated by Equation (11).
